# Population cardiovascular health and urban environments: the Heart Healthy Hoods exploratory study in Madrid, Spain

**DOI:** 10.1186/s12874-016-0213-4

**Published:** 2016-08-22

**Authors:** Usama Bilal, Julia Díez, Silvia Alfayate, Pedro Gullón, Isabel del Cura, Francisco Escobar, María Sandín, Manuel Franco, the HHH Research Group

**Affiliations:** 1 Social and Cardiovascular Epidemiology Research Group, School of Medicine, University of Alcalá, Alcalá de Henares, Madrid, 28871 Spain; 2 Department of Epidemiology, Johns Hopkins Bloomberg School of Public Health, Baltimore, MD USA; 3 Unidad Docente Medicina Preventiva y Salud Pública, National School of Public Health, Madrid, Spain; 4 Primary Care Research Unit. Gerencia de Atención Primaria, Madrid, Spain; 5 Department Preventive Medicine and Public Health, University Rey Juan Carlos, Madrid, Spain; 6 Red de Investigación en servicios sanitarios en enfermedades crónicas (REDISSEC), Madrid, Spain; 7 Department of Geology, Geography and Environment, Faculty of Biology, Chemistry and Environmental Sciences, University of Alcalá, Alcalá de Henares, 28871 Madrid Spain

**Keywords:** Cardiovascular disease, Residential environment, Neighborhoods, Mixed methods, Spain

## Abstract

**Background:**

Our aim is to conduct an exploratory study to provide an in-depth characterization of a neighborhood’s social and physical environment in relation to cardiovascular health. A mixed-methods approach was used to better understand the food, alcohol, tobacco and physical activity domains of the urban environment.

**Methods:**

We conducted this study in an area of 16,000 residents in Madrid (Spain). We obtained cardiovascular health and risk factors data from all residents aged 45 and above using Electronic Health Records from the Madrid Primary Health Care System. We used several quantitative audit tools to assess: the type and location of food outlets and healthy food availability; tobacco and alcohol points of sale; walkability of all streets and use of parks and public spaces. We also conducted 11 qualitative interviews with key informants to help understanding the relationships between urban environment and cardiovascular behaviors. We integrated quantitative and qualitative data following a mixed-methods merging approach.

**Results:**

Electronic Health Records of the entire population of the area showed similar prevalence of risk factors compared to the rest of Madrid/Spain (prevalence of diabetes: 12 %, hypertension: 34 %, dyslipidemia: 32 %, smoking: 10 %, obesity: 20 %). The food environment was very dense, with many small stores (*n* = 44) and a large food market with 112 stalls. Residents highlighted the importance of these small stores for buying healthy foods. Alcohol and tobacco environments were also very dense (*n* = 91 and 64, respectively), dominated by bars and restaurants (*n* = 53) that also acted as food services. Neighbors emphasized the importance of drinking as a socialization mechanism. Public open spaces were mostly used by seniors that remarked the importance of accessibility to these spaces and the availability of destinations to walk to.

**Conclusion:**

This experience allowed testing and refining measurement tools, drawn from epidemiology, geography, sociology and anthropology, to better understand the urban environment in relation to cardiovascular health.

**Electronic supplementary material:**

The online version of this article (doi:10.1186/s12874-016-0213-4) contains supplementary material, which is available to authorized users.

## Background

Cardiovascular diseases (CVD) remain the leading cause of death worldwide [1]. Their burden is projected to escalate in the following decades due to increased prevalence [2]. The large costs associated with CVD fall both on the social and economic side and the lack of effective preventive measures will make these costs difficult to deal with for governments worldwide [3, 4]. Individual risk factors directly associated with CVD include behavioral traits as smoking, unhealthy diets, lack of physical activity and excessive consumption of alcohol [5]. These behavioral risk factors and their associated increases in biological risk factors as hypertension, dyslipidemia and diabetes represent a large proportion of the excess CVD risk in populations. In particular, it has been estimated that there’s an opportunity to prevent even more CVD deaths in Spain if we can curb the increase in some risk factors such as diabetes [6].

Prevention efforts are much needed to continue decreasing the incidence of CVD. The population preventive approach [7] has previously shown large reductions in CVD, either through well-designed whole population campaigns [8] or through large political or economic changes [9]. This approach has a large potential preventive effect since it tackles the root causes, which are mostly social, political and economic [10], of the distribution of chronic diseases in a given population. One of the social units that may better exemplify whole population preventive strategies are urban neighborhoods [10]. Public health research at the neighborhood level tries to characterize which features of the local residential environment are key in the distribution of disease risk among populations. Methodological advances in the last decades, such as multilevel modeling [11], have allowed for simultaneous analysis of individual and contextual effects, removing much of the limitations of individual or ecological based analysis.

At the same time the growing use of electronic health records (EHRs) offers a tremendous opportunity to public health researchers to measure residents health outcomes [12] by neighborhood. Results from these EHRs studies will expand the evidence to improve cardiovascular health at a population level.

In terms of being able to fully characterize the urban environment [13], borrowing methodologies and techniques from social sciences such as geography are key. Current attained level of development of Geographic Information Systems (GIS) has made possible relevant advances in this area.

However, previous research has shown that objective neighborhood resources are not always consistent with residents’ perceptions. Qualitative methods, such as semi-structured interviews, enable the examination of this complex association between neighborhoods and the impact on residents’ health outcomes. This combined use of different perspectives and methodologies has been recently defined as mixed-methods research [14], focusing on research questions that call for real-life contextual understandings, multi-level perspectives, and cultural influences [14, 15].

**Fig. 1:**
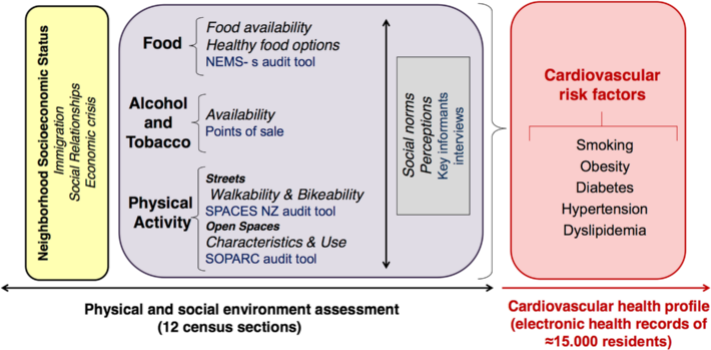
Conceptual framework of this study. The environmental outcomes assessed are shown in italics, whereas the type of measurement are shown in blue. The cross-cutting approach of the qualitative methodology is highlighted throughout the grey box

This is an exploratory study framed within a larger study, the Heart Healthy Hoods [10, 16], aiming at characterizing the entire city of Madrid (Spain) and the cardiovascular health of its residents. A photographic depiction of the study area of the present manuscript can be found elsewhere [16] (the middle income area). Results from this experience can help other researchers design urban health studies that completely characterize a residential environment and the health of its residents. We aim to fully characterize an urban area using several measurement tools and approaches, basing our strategy on a theory-driven approach shown in Fig. 1. As proposed by Sacristan [17], we started with a theory-driven framework where we will explore its feasibility and add new hypotheses as a result of this exploratory study.

In the spirit and recommendations of Thabane et al. [18], we do not present any hypothesis testing results, but rather leave open several questions for future research in the main study. This is also in concordance with the approach proposed by Shankdardass and Dunn [19], who advocate for more “intensive” neighborhoods research, as opposed to “extensive” research. In summary, extensive research seeks to draw inferences about the quantification of neighborhood effects in the “general” population of neighborhoods. Intensive research instead seeks to uncover *how* neighborhood effects work and what are the best points of action to affect them.

The objective of this study is therefore to: (a) describe the cardiovascular health profile of a population over 15,000 residents living in this area analyzing the Madrid Primary Health Care System electronic health records; and (b) explore different quantitative and qualitative measurements to characterize the social and physical urban environment in relation to food, alcohol, tobacco and physical activity.

## Methods

### Study design and setting

**Fig. 2:**
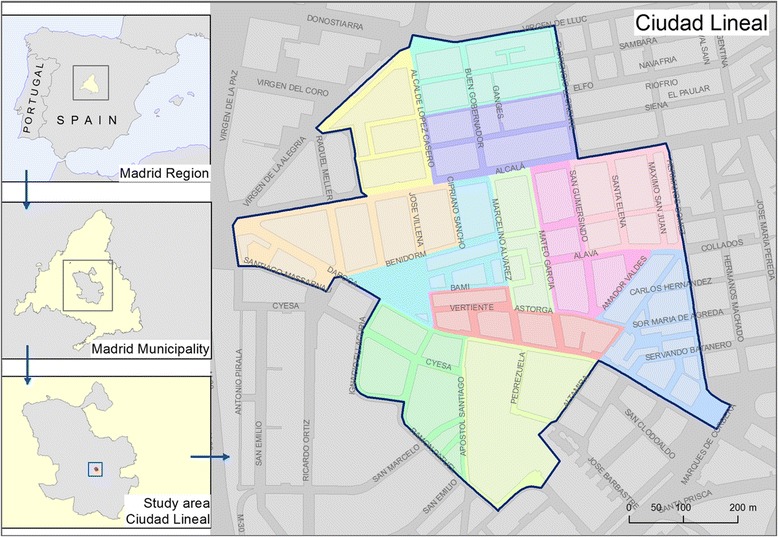
Heart Healthy Hoods exploratory study setting (12 census sections in Ciudad Lineal, Madrid, Spain)

This is an exploratory study conducted in 12 contiguous census sections of Madrid (Fig. 2) between March 2013 and June 2014 describing the Cardiovascular Health profile and Risk Factors of its residents and the social and physical urban environment in which they live. In order to conduct our study in an area that was not extreme in sociodemographic or urban form terms, we selected these 12 census sections using the Median Neighborhood Index. This method selects clusters of census sections that are on average closest to the median neighborhood in four variables: % above 65 years of age or older, % with low education, % foreign-born and population density. More details on this method can be found in the Additional file 1: S1.

### Quantitative measurements of cardiovascular health and risk factors

Spain’s National Health System (SNS) is publicly funded, providing universal health care coverage free of charge at the point of use. The National Health System structure is region-based, and organized into health areas and basic health zones. Electronic health records share the same system and software at the region-level. These records include diagnoses for conditions such as diabetes or hypertension that have been previously validated [20, 21] and other diagnoses such as dyslipidemia, obesity or smoking.

The study population was restricted to those individuals aged 45 and above, holding a health care identification card and living within the 12 census sections. Cardholders needed to have visited their primary care health center in the last year at least once prior to the data mining. We collected anonymized data from electronic health records on cardiovascular health and risk factors (tobacco use, obesity, hypertension, diabetes mellitus, dyslipidemia) and sociodemographic variables (age, sex). In all cases of diabetes, hypertension and dyslipidemia, the diagnoses were physician-based. Obesity was assessed by computing BMI (kg/m^2^) from the last available measure of height and weight and was defined as a BMI > =30 kg/m^2^. Smoking was assessed by asking individuals about current cigarette smoking. According to the internal primary care guidelines, all individuals aged 14 or above should have at least two of the risk factors mentioned above (plus sedentarism and alcohol consumption) measured in the previous 5 years. Moreover, all individuals without prevalent cardiovascular disease or diabetes and aged between 40 and 65 (which includes our study population) should get their cardiovascular risk assessed every 2 to 5 years (for medium/high and low risk individuals, respectively). This cardiovascular risk assessment includes the measurement of blood pressure and lipids and assessing tobacco use (as described above). Anonymization was conducted by removing all personally identifiable information (address, name, identifiers) and aggregating the results to the census section level.

### Quantitative measurements of the urban environment

We selected audit tools from other countries (mostly the US and Australia) given the scarcity of studies measuring specific characteristics of urban environments in Spain. All audit tools selected below were selected based on their simplicity and similarity to Spanish urban environments. When possible, we elected to do the fewest amount of adaptations possible to improve comparability with other international studies.

#### Food environment

We identified all food stores in the area by direct observation. We classified and conducted a direct auditing of all food stores present. Classification was done ad hoc following Table 1. This classification, which relates to the size, and range of food options available at the food stores, follows the categorization used by the Nutrition Environment Measurement in Stores (NEMS-S) [22]. A trained data collector conducted direct auditing of all food stores following a brief version of the NEMS-S tool (For the brief instrument and the adaptations see Additional file 1: S2). We then computed a Healthy Food Availability Index for each store following the scoring system in the Additional file 1: S2. This HFAI score ranges from 0–28, with a higher score indicating a greater availability of healthy foods. We also assessed public markets in the area and classified each stall as either a specific specialty store (e.g.: fruit/vegetable) or a small grocery store (selling a variety of items). Public markets in Spain are a collection of tens of stalls (in our case, more than 100) mostly dedicated to retailing a single category of foods (e.g.: fruits/vegetables, fish, meat, bakery products, etc.). For this reason and considering that the NEMS-S was designed around measuring scattered discrete stores, we decided not to compute a Healthy Food Availability Index for the public market and just describe the number and type of stalls.

**Table 1:** Classification and description of food store types

Type of Store	
Public Market	Municipally owned building where vendors sell fresh food from open stalls.
Supermarket	Large corporate owned “chain” food stores with several employees and cash registers.
Small Grocery	Non-corporate-owned small food stores, with no more than 1 cash register.
Specialty Store	Small food store that sells only one group of foods (eg: fruits/vegetables, butchers, fishmongers)
Corner Store	Small store with long shopping hours and (generally) owned by ethnic minorities.
Convenience Store/Gas Station	Food stores with a limited selection of foods, with long shopping hours (>18 h/day), attached or not to a gas station.

Food services (restaurants, bars, fast food options, etc.) were classified into fast food restaurants and sitting down restaurants using the same classification as the Nutrition Environment Measurement in Restaurants (NEMS-R) inventory [23].

#### Alcohol and tobacco environment

We identified all tobacco and alcohol outlets in the area by direct observation (analogous to the observation of food stores). We characterized the tobacco and alcohol environment by classifying all retail outlets that sold either tobacco or alcohol into the following categories: tobacco stores and vending machines; bars or restaurants (selling alcohol for consumption on-site), food stores selling alcohol (a majority of the food stores present in the area) and liquor stores. Spanish law heavily regulates retail sales of tobacco, that can only be conducted through tobacco stores (called “Estancos”) or vending machines, which have to be located in establishments previously authorized from the *Commissioner for the Tobacco Market* (such as newspaper stands located on public roads, certain convenience stores or bars and restaurants). The number of vending machines per area is also regulated and is linked to the number of tobacco stores in the area.

#### Physical activity environment

We measured two aspects of the physical activity environment, the characteristics of streets and the use of open spaces. To characterize streets, we used the Systematic Pedestrian and Cycling Environment Scan (SPACES) [24], an observational audit of urban infrastructure that can influence walking and cycling [25] and that has been validated in Madrid [26]. We collected information on every street segment of the study area (*n* = 152) for the four SPACES factors: function, safety, aesthetics and destinations. We have previously published more details on this procedure and its measurement properties (reliability and validity) in Madrid [26]. In order to measure the use of parks and open spaces within and next to the study area, two field researchers completed the System for Observing Play and Recreation in Communities (SOPARC) instrument [27] in all parks and open spaces of the area (identified through direct observation, *n* = 10). The two researchers stood on a pre-specified location of the park and observed park usage for 1 h. Every individual using the park was observed and classified regarding basic sociodemographic characteristics (age, gender, ethnicity) and type of park use regarding levels of activity (sedentary, walking or vigorous).

### GIS-based data integration

Aiming at the implementation of a comprehensive geo-referenced database of the pilot study area, we collected information from the following sources:

- Spanish National Spatial Data Infrastructure (IDEE), National Mapping Agency (IGN): line and polygon vector layers such as street sections, administrative boundaries and blocks.
- Madrid Regional Spatial Data Infrastructure: point vector layers on retail stores, restaurants and gas stations.

These layers were loaded into ArcGIS 10.01 and projected to a common system (ED50 UTM 30). Fieldwork results on both street-based and Google Street Map-based audits were then joined to the street sections layer by means of relational union. All other layers (different types of administrative boundaries and blocks) were introduced to the final maps as reference information.

### Qualitative interviews on the urban environment

In order to provide insights and to improve the understanding of our quantitative findings, we performed a second assessment of the area through qualitative methods. We conducted a series of semi-structured interviews with key informants (according to the sociodemographic structure of the area, including age and ethnicity, and the domains we wanted to gather information about) that had lived in the area for a long time, choosing information-rich cases selected using stratified purposeful sampling [28]. We included the following 11 key informants: a health care provider (female), the director of the health promotion center of the area, a local food store owner, four local residents (two females and two males, 45–65 years and > 65 years respectively), two immigrants (female and male), one primary school teacher and one community activist. These interviews included general questions about health and the environment and more focused questions about the neighborhood sociodemographics, neighborhoods boundaries, their individual perception on environmental characteristics and social norms regarding food, physical activity, alcohol and tobacco. Analysis of the interviews was carried out by three researchers following the validity criterion of investigator triangulation [29] and according to the steps of analysis in progress [30], incorporating an interpretative phenomenological analysis [31] perspective.

#### Mixed method approach

In this exploratory study we decided to combine the different quantitative and qualitative data, following a merging data approach [14, 32] presented in the result and discussion sections. Our objective with this merging phase was two-fold: (a) to provide insights on the phenomena behind our quantitative findings; and (b) to use qualitative research as a formative research phase that would guide our future data collection.

## Results

### Cardiovascular health profile and risk factors results

Fourteen and eight hundred fifty-seven thousandths residents of the study area are holders of a Health ID card and are assigned to one of the three Primary Care Centers present in the area. This represents 96.3 % of the 15422 residents living in the study area according to the municipal registry. The average age of this population was 45 years and 55.1 % were female. Table 2 shows the total prevalence of cardiovascular risk factors by gender in the population of the study area aged 45 and older. About 12 % of the population above 45 had diagnosis diabetes, 32 % had a diagnosis of dyslipidemia, 34 % had a diagnosis of hypertension, 10 % reported current smoking and 20 % were obese.

**Table 2:** Population cardiovascular health profile and risk factors of the residents aged 45 and older in the study area

Prevalence (%)	Men	Women	Total
Diabetes	14.0	10.0	11.6
Dyslipidemia	27.4	34.8	31.8
Hypertension	30.2	36.2	33.7
Smoking	12.6	7.9	9.8
Obesity	17.3	21.5	19.8

Diabetes, dyslipidemia and hypertension are physician diagnosed. Smoking is defined as current vs. not smoking. Obesity was defined as a BMI > = 30 kg/m^2^, computed from the last available measure of height and weight

### Food environment

**Fig. 3:**
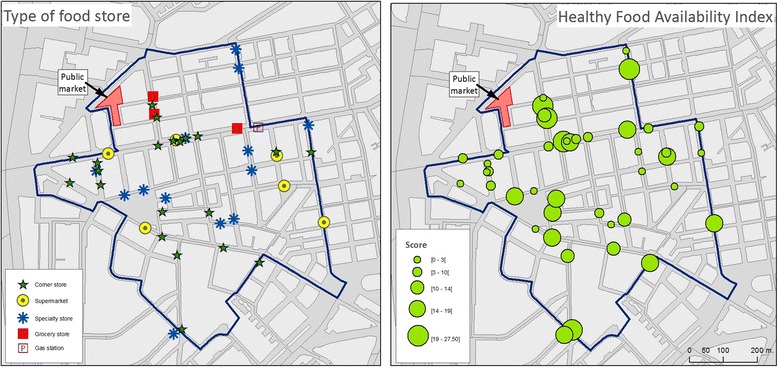
Food environment results in the study area (12 census sections), including type of food stores (*left*) and their healthy food availability index scores in quintiles (*right*)

**Fig. 4:**
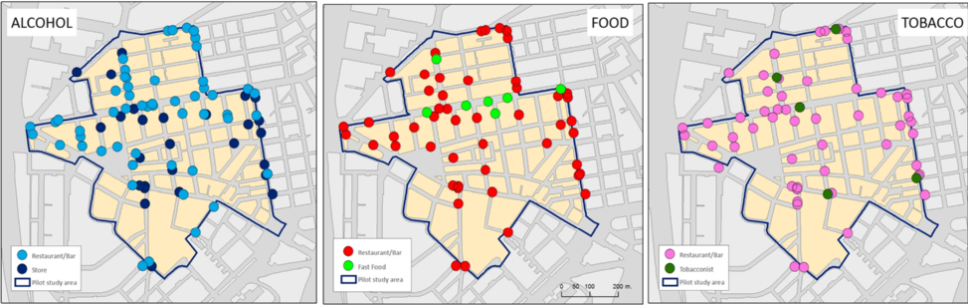
Alcohol (*left*), Food Services (*middle*) and Tobacco (*right*) environments in the study area

Forty-four food stores were located in the study area (Fig. 3). Supermarkets scored highest in terms of Healthy Food Availability (25.5 out of 28) and convenience stores the lowest (7.5 out of 28). Two food markets (the “Las Ventas” and “Bami” markets) were present in the area. The “Las Ventas” public market is a 3-storied indoors market with 112 stalls (most of them selling fruits/vegetables, meat/dairy or fish). There were 61 food service business present (Fig. 4) and most of them (*n* = 53) were regular sitting down restaurants.

Qualitative results showed several important concepts: the concept of affordability, where high quality and healthier food options are perceived as more expensive; and the concept of “distance to stores”, which is also believed to be an important determinant for accessibility, especially for the elderly.

“*I have my children and many years, so I know what is good and what is bad…what one can afford is different” (woman, >65 years)*

Interviews also highlighted the importance of the concept of “lifetime store”, owned by local people that have a long history of dealing with neighbor’s needs and trust.

### Alcohol environment

The alcohol environment in the area is very intertwined with the food environment. All but one of the 91 alcohol outlets in the study area (Fig. 4) was also either a food store (only 1 of the 32 off-sale alcohol outlets was a liquor store) or as a bar/restaurant (all 59 alcohol on-sale outlets). Qualitative interviews showed that alcohol consumption is believed to be mostly influenced by individual choices rather than the social environment. Besides, there is a perception that excessive alcohol consumption has low prevalence. The alcohol environment is also linked to socialization, with positive connotations, but perceived to be affected by the economic crisis:

*“I get along well (with neighbors); I drink beer with whomever I want to” (man, <65 years).*

*“Social drinking customs are disappearing, we used to go on Sundays to have a vermouth with your neighbors and your friends. Nowadays, people are doing it less, because of the economic crisis” (Food store owner).*

### Tobacco environment

There were 64 tobacco outlets in the area. Of these, 6 were exclusive tobacco outlets and 58 were automatic vending machines located in bars or restaurants (and therefore sharing space with food and alcohol retailing) (Fig. 4). As seen below (Fig. 4), tobacco outlets or vending machines are ubiquitous within the area. Interviews revealed contradictions regarding trends in smoking prevalence: smokers perceive that the local availability of tobacco remains stable while non-smokers perceive the opposite.

### Physical activity environment

**Fig. 5:**
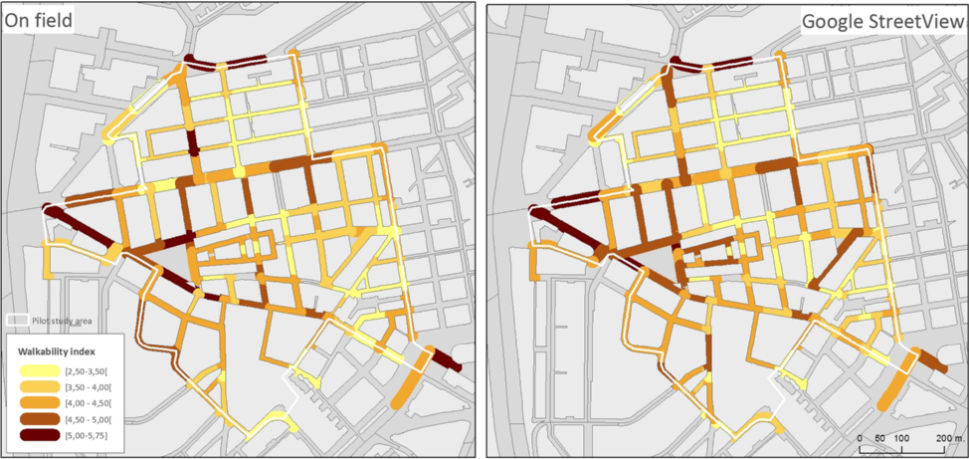
Walkability index in the study area, on-field visits (*left*) and Google Street View (*right*)

The walking environment showed heterogeneity around the study area (Fig. 5). The two main avenues of the study area (*Calle Alcala* and *Avenida Daroca*) had the highest scores for walkability, especially due to the size of their sidewalks and the presence of a large amount of destinations. Qualitative research highlighted architectural barriers influencing mobility patterns of elder residents:

*“When we are older, because I’m on a wheelchair in the street … If I had benches there, I would not need the wheelchair, because walking 20 m is fine, but maybe 25 m isn´t.” (Woman, > 65 years)*

Regarding open spaces and parks, the results from the SOPARC instrument show that the majority of users of all parks were male (66 % of all park users, a majority in all 10 parks but one) and adult or seniors (64 and 17 % of all park users, respectively). The level of activity varied each open space: in 4 of them the main level of activity was sedentary, in 2 the main use was for walking and in 4 was there was a majority of people doing vigorous physical activity. Contrary to our observations, interviews with residents highlighted the more intense use of parks by young immigrants or minorities. Moreover, neighbors also expressed reluctance to use these open spaces where the proportion of immigrant people was high:

“*There have been parks that have been taken over by gangs of immigrant kids; at certain times one is afraid of passing through; even as an adult” (Man < 65 years).*

### Emerging results from qualitative in-depth interviews

The analysis of these 11 interviews showed four important emergent categories: 1) the individualized definition of neighborhood boundaries, 2) the effect of the current economic crisis on neighbors’ behavior, 3) the role of immigration, and 4) the importance of social relationships in neighborhood use (See Table 3). The economic crisis is a cross-sectional element in the discourse of the interviewees.

**Table 3:** Emerging categories in in-depth interviews

Neighborhood boundaries are subjective.
*“We take a compass and put the center of the compass (from his home) to Quintana and the circle is round. That would be my neighborhood “*(Food store owner)
Economic crisis influences neighbors behavior.
*“… Nowadays there are a lot of grandparents taking care of the family…. Many unemployed descendants. So there is little time for healthy habits like exercise… ” (health care provider, woman)*
Immigration is seen as very influential element in neighborhood life.
*“… In the past other people would go there [park], but now the Romanians are there…” (men, < 65 years)*
Social relationships affect the use of the neighborhood.
*“I’m happy with people in my neighborhood. Since my husband died, … adults and kids alike, boys like my sons, 50 years-old, [have told me] “hey, I work on this, if I can help you… I will help you with stuff if you ask me””* (woman, > 65 years)

## Discussion

This study allowed us to test the feasibility of doing an in-depth study of a neighborhood and its environmental and social determinants of cardiovascular health. Through a series of quantitative and qualitative techniques we were able to measure different aspects pertaining to cardiovascular health that were included in our framework: the food, physical activity, tobacco and alcohol environments, and habits and social norms related with them. By using the electronic health records of the Universal Primary Health Care system we were able to obtain a picture of the cardiovascular health of the residents in the area. We drew methods from epidemiology, geography, sociology and anthropology, and combined them to make the best possible characterization of a neighborhood cardiovascular environment.

Cardiovascular health in the area was similar to the Madrid total population in terms of prevalence of cardiovascular risk factors such as hypertension, diabetes and dyslipidemia. The validity of electronic health records as methods to estimate prevalence has been shown for hypertension and diabetes in Madrid [20]. Smoking prevalence was lower in electronic health records compared to population surveys, potentially reflecting underreporting of smoking prevalence in primary care. Future work should emphasize the need for a more systematic validation of electronic health records data (see Table 4). One of the main advantages of using electronic health records of a universal primary care health system is the feasibility to scale up the measurement, that, in the case of Spain, can be done up to the regional level (Madrid Region, more than 6,000,000 people). These measurements are available down to a small scale (census sections, around 1000 people) and allow for small area comparisons similar to what has been done in studies in the US [33] or the UK [34] or even Spain for mortality [35]. Spain has almost universal coverage of public insurance (>99 %) and we were able to ascertain that we had data on more than 96 % of the people living in the area (according to the municipal registry). The use of these systems for continuous chronic disease surveillance (see Table 4) will increase opportunities for prevention, as seen in cases like New York [36].

**Table 4:** Conclusions of the Heart Healthy Hoods exploratory study: challenges and opportunities for measuring urban environments and cardiovascular health

	Quantitative measurements	Qualitative measurements	Geographic Information Systems
Electronic Health Records	Validation of EHR diagnosis (beyond diabetes and hypertension).	Not available. Can be performed in a selected subset.	GIS allows for data integration of location and attributes of features of each domain, administrative boundaries, public transportation network, parks and street segments. With this data integration, geospatial analysis of various kinds can be performed. Future data should include accessibility, other distance-based indicators, the use of more detailed geostatistics (dispersion, centrality, etc.) and other tools (such as map algebra). Availability of sufficient quality data. Design and validation of a cartographic model, based on a combination of the above analyses, to produce meaningful composite indices.
	Use of EHR for continuous surveillance of chronic diseases.		
Food environment	More emphasis should be placed on the measurement of affordability.	A more in-depth approach to dietary patterns is needed.	
	A further culturally adapted NEMS-S survey is needed.	Better insights to the effects of family composition on dietary patterns.	
	Public markets are a unique feature in Spain.		
Alcohol environment	Use of implementation science tools to measure compliance.	Further exploration of spaces of consumption and social norms associated to these.	
Physical activity environment	Validation of virtual audit methods (Google Street View)	More in-depth insights on barriers to physical activity (including physical and social barriers)	
Tobacco environment	Measurement of exposure to second-hand tobacco.	Perceptions regarding smoking need to be stratified by smoking status.	
	Use of implementation science tools to measure compliance with tobacco regulations	More research is needed on social norms that influence smoking and the implementation of smoking regulations	

On the side of the exposure, in this case urban environments, we found a very rich food environment. An important challenge we found in this exploratory study was the measurement of public markets. The area had two of these, one of them with long opening hours and more than 100 stalls. Standard tools for healthy food availability measures (like our abbreviated NEMS-S tool) can fail to capture the effect of these type of retailers (see Table 4). A second challenge is the lack of an appropriate food affordability measure. Interviews with neighbors showed that prices determine what people can afford and therefore the food and that they can buy (see Table 4). Moreover, they also expressed concern for the lack (or availability) of ethnic foods. Affordability and cultural acceptability are two of the four key aspects of the local food environment (with the other two being accessibility and availability, measured with our current tools) [37, 38]. Therefore we need to adapt tools such as Market Basket Surveys to the Spanish context (see Table 4) [39].

The alcohol and tobacco environment was mostly dominated by bars and restaurants. There were only 5 exclusive tobacco stores (heavily regulated in availability and prices by the government) and only one exclusive liquor store. Every other retail business for tobacco was either a bar or a restaurant, coinciding with on-sale alcohol outlets (where alcohol is consumed on site) that also provide food services. This combination, in a single business, of on-sale alcohol outlets, tobacco automatic selling machines and food services are a staple business in Spanish neighborhoods and are therefore one of the most relevant components of the food, alcohol and tobacco environment in Spain. Most research conducted outside of Spain focus on specific off-sale outlets that are specialized in alcohol retailing. Our tools did not capture off-sale alcohol availability in food stores (supermarkets, corner stores, grocery stores) but these are the most commonly used points of sale for alcohol in Spain. Future re-designing of these tools must incorporate this intertwined nature of the food and alcohol environment. Interviews with neighbors showed the cultural importance of alcohol consumption in these bars as a social cohesion mechanism. In other contexts alcohol outlet density has been related to alcohol consumption and crime before [40], but we are not aware of similar research conducted in Spain. We are currently exploring other alcohol environment measures that may vary more by context, like marketing and advertisement outside of bars and restaurants. Tobacco outlet density has been linked to tobacco consumption or reduced chances of tobacco quitting [41], but the availability of exclusive tobacco stores is heavily regulated by the government in Spain. Tobacco sales in bars/restaurants in Spain happens under automatic vendor machines. There is exiting data on the location (and sales) of these machines, gathered by the regulatory commission of tobacco in Spain. After several requests (for research purposes), we have not been able to obtain such data for unclear “economic” reasons.

In the physical activity environment most open spaces were used by adults, especially seniors without a clear intent to engage into physically active. This may be due to the design of these open spaces as more than two thirds of the open spaces did not have a design conductive for anything but walking or passive use. Interviews with neighbors showed an interesting duality regarding preferred places to walk: while parks were well perceived, their use is conditioned on the presence of certain behaviors (such as alcohol consumption or immigrant presence), and some people preferred walking in streets with a high density of retail business, rather than walking on parks or other open spaces. While some of our tools were able to capture these characteristics, they were resource-intensive and required prolonged times of observation. We validated the SPACES audit tool for walkability measurements using Google Streetview [26] (see Table 4), but found that virtual measurement time was analogous to on-field measurement time (with only the advantage of not having to travel to the study area). We are now exploring and validating measurements of walkability that do not require extensive audits and leverage the power of GIS [42].

The integration of all collected data using Geographic Information Systems is an opportunity to accommodate the different domains that make up a given urban environment. GIS also allows for geospatial analysis and the construction of more detailed indicators. Two main challenges resulted from this exploratory study: (a) the development of meaningful composite indices that combine the study domains; and (b) the integration of the temporal dimension, including business hours (for accessibility) [43] and activity time-spaces of the residents [44] (see Table 4).

The qualitative part of our mixed methods approach let us to get a clear picture of the area from the “experts”, that is, the neighbors that live in them. These methods allow vulnerable populations (that may not be covered in quantitative studies) to get a voice in research [45]. Semi-structured interviews allowed us to get access to individual perceptions, but proved to be less useful in topics like alcohol and tobacco (see Table 4). Given the intense social component of alcohol and cigarette smoking we believe that methods like focus groups [46] or concept mapping [47] may be more useful. Moreover, we were also able to uncover the different levels at which neighbors perceive that the environment affects them. While, as mentioned above, neighbors perceived that smoking was less affected by neighborhood characteristics, neighbors remarked the importance of national level (macro) policies in reducing smoking prevalence. Moreover, while neighbors did not perceive that the local environment influenced alcohol consumption, they did emphasize the importance of social interactions (micro) and drinking. Food and, more importantly, physical activity, were domains in which neighbors did perceive strong influences of their local environments. Being cognizant of the levels at which each health outcome is determined is an important task in neighborhoods effects (and other social epidemiologic) research.

The combination (in our case, concurrent integration) of qualitative and quantitative data through a mixed methods approach is an adequate approximation to complex social phenomena [14]. This concurrent integration approach to merging quantitative and qualitative data increases understanding or develops a complementary picture; nonetheless, we also believe that a sequential timing approach (e.g.: an initial phase of formative qualitative research followed by the design of quantitative tools) would have helped us in avoiding some of the pitfalls described in this manuscript. We acknowledge that mixed method approaches have their own difficulties, like the scarcity of a training infrastructure, the necessity to work under two epistemological traditions or the complexity of data integration [48, 49]. Nonetheless we believe they remain a useful approach to study neighborhoods where “the whole is greater than the sum of the parts” [50].

## Conclusions

This experience allowed testing and refining measuring tools to understand neighborhood characteristics in relation to cardiovascular health (See Table 4 for a complete list of future challenges and opportunities). Several quantitative epidemiological and geographical methodologies showed to be complementary and relevant when describing the specific features of the urban environment. The inclusion of qualitative methodologies provided important insights adding emergent categories to the characterization of neighborhoods such as: subjective neighborhood boundaries, the effect of the economic crisis on businesses and on neighbor’s consumption patterns, the importance of social networks and the relevance of immigration in neighborhoods life. The combination of urban environment measurements, quantitative and qualitative, and universal electronic health records from the primary care health system, will provide useful data to examine the relationship of neighborhood characteristics and cardiovascular health shedding important light to develop sound population preventive approaches.

## Additional file

Additional file 1:
**S1.** The Median Neighborhood Index Methodological Details. **S2.** Adapted NEMS-S Audit Tool. (DOCX 137 kb)

## Abbreviations

CVD: Cardiovascular diseases
EHR: Electronic health records
GIS: Geographic information systems
HFAI: Healthy food availability index
NEMS-R: Nutrition environment measure survey-restaurants
NEMS-S: Nutrition environment measure survey-stores
SOPARC: System for Observing Play and Recreation in Communities
SPACES: Systematic pedestrian and cycling environment scan
UK: United Kingdom of Great Britain and Northern Ireland
US: United States of America.
