# Supplementary material for: Population cardiovascular health and urban environments: the Heart Healthy Hoods exploratory study in Madrid, Spain
Source: BMC Med Res Methodol. 2016 Aug 22;16(1):104. doi: 10.1186/s12874-016-0213-4 (PMC4994419; doi:10.1186/s12874-016-0213-4)
Supplement: Additional file 1: — S1. The Median Neighborhood Index Methodological Details. S2. Adapted NEMS-S Audit Tool. (DOCX 137 kb) [file 12874_2016_213_MOESM1_ESM.docx]

**S1: The Median Neighborhood Index Methodological Details**

**The Median Neighborhood Index**

The Median Neighborhood Index (MNI) is the average Euclidean rank distance of each spatial unit of analysis to the median neighborhood in a series of variables. More specifically, this index uses four variables to represent the demographic and socioeconomic structure, segregation phenomena and urban form. The Euclidean rank distance in each variable is calculated by sorting all units of analysis (census sections) and computing how far in rank each unit is from the median neighborhood. The four distances are then averaged to the Median Neighborhood Index. A low value in this index represents a more average neighborhood in the four variables, while a higher value represents more extreme neighborhoods. Importantly, and since rank distances are all positive, these extreme neighborhoods may be on either tail of the distribution of social factors.

**Variables**

For the four variables, we used % population aged 65 or above as the demographic indicator, % people with college education or above as the socioeconomic indicator, % foreign-born as the segregation indicator, and population density (in sq. km) as the urban form indicator. The unit of analysis was the census section (around 1500 people).

**Selecting Average Neighborhoods**

To select average neighborhoods we look for clusters of spatial units of analysis of the desired size. For example, if the unit of analysis is the census sections and we seek an area of 15,000 people, we must seek clusters of a maximum of 12 census sections. We use Kulldorf’s Spatial Scan Statistic (Kuldorff 1997). This method allows for the search of clusters of cases, normally distributed variables or other distributions. Given the normally distributed nature of the MNI, we looked for clusters of low MNI values. The Kulldorf’s Spatial Scan Statistic also allows for the setting of a maximum cluster size. Given that this statistic requires for spatial point data to be used, we calculated the centroids of each spatial unit of analysis prior to the cluster search.

**S2: Adapted NEMS-S Audit Tool**

**Adaptations (from the Abridged NEMS version produced by the CLF [available at** [**http://mdfoodsystemmap.org/)**](http://mdfoodsystemmap.org/))**:**

- +1 For Ground beef available changed to +1 For any ground meat
- +1 for Lean ground beef changed to +1 for beef
- .5 for frozen fruits or vegetables changed to +1 for each
- +1 if any of dried beans, rice or pasta available, changed to +0.5 for each
